# Sciatic nerve injury treated by acupuncture: a bibliometric study and visualization analysis

**DOI:** 10.3389/fneur.2024.1432249

**Published:** 2024-12-31

**Authors:** Yan Wang, Yan Wang, Fei Pei, Yahui Wang, Lijie Lv, Tianyi Li, Shujin Wang, Huan Qin

**Affiliations:** ^1^The Second School of Clinical Medicine, Heilongjiang University of Chinese Medicine, Harbin, China; ^2^Rehabilitation Center, The Second Affiliated Hospital of Heilongjiang University of Chinese Medicine, Harbin, China

**Keywords:** sciatic nerve injury, acupuncture, visualization analysis, VOSviewer, CiteSpace

## Abstract

**Objective:**

Sciatic nerve injuries often lead to severe pain and motor dysfunction, causing serious impact on patients’ quality of life. Acupuncture, as one of the main therapies in traditional Chinese medicine, is gradually gaining attention in the field of nerve injury due to its potential role in pain relief and nerve repair. Bibliometric and scientific knowledge mapping methods were employed to analyze the current research status, hotspots, and development trends of acupuncture for sciatic nerve injury (SNI) over the past decade.

**Methods:**

A literature search was conducted on acupuncture for SNI from the China Knowledge (CNKI), Wanfang, Weipu (VIP), and Web of Science (WOS) databases, and CiteSpace V6.R6 software and VOSviewer1.6.20 software were employed to analyze and visualize keywords from the collaborative network, keyword co-occurrence, and keyword timeline, respectively. Keyword emergence was analyzed and visualization maps were drawn.

**Results:**

A total of 907 articles were included in the Chinese database, while 78 articles were included in the WOS database. The leading institutions of the literature in Chinese and English were the Liaoning University of Traditional Chinese Medicine and the Institute of Acupuncture and Moxibustion, respectively. Ma Tieming is the most influential person in the field. A keyword analysis of the Chinese and English literature revealed that Huanjiu and Huizhong points are commonly used and that acupuncture is often applied with drug therapy, physical therapy, and rehabilitation training, which can improve the treatment effect comprehensively. Additionally, the therapeutic mechanism of acupuncture mainly promotes nerve regeneration and repair through the modulation of cytokines and related signaling pathways. Electroacupuncture and neuropathic pain are cutting-edge research topics in this field. Based on the keyword emergence, it is predicted that future studies will continue to focus on acupuncture treatment, functional recovery after SNI, and spinal cord injury, which may become the research hotspots in this field. In addition, future studies could further delve into the selection and physiological mechanisms of acupuncture points and validate the effectiveness of this acupuncture and its integrative therapeutic strategies through a wider range of clinical practices.

**Conclusion:**

This study uses visualization software VOSviewer and CiteSpace to provide the current status and trends in SNI of acupuncture over the past decade and predicts potential research frontiers and hot directions.

## 1 Introduction

Sciatic nerve injury (SNI), a common peripheral neuropathy, is usually caused by disc herniation, pelvic or lumbar trauma, and other factors (1, 2). This lesion is clinically characterized by pain, paresthesias, and dysfunction (3). Patients typically experience severe low back and hip pain that often radiates down the nerve pathways to the legs, severely impacting their quality of life (4). Although medications and physical therapy have been effective in treating SNI, they are often associated with side effects and risk of recurrence. Therefore, the exploration of safer and more effective alternative treatment strategies has become an important research direction in this field. Acupuncture, as a non-pharmacological therapy, has gradually gained attention in clinical practice. It is preferred because of its unique therapeutic mechanism and lower risk of side effects (5). According to studies, acupuncture can promote the overall recovery of patients by promoting blood circulation, regulating the nervous system and inflammatory factors, and improving nerve conduction, among other mechanisms (6).

CiteSpace and VOSviewer are two specialized tools used for analyzing and visualizing scientific literature networks. In the analysis of literature on acupuncture treatment for sciatic nerve injury, CiteSpace particularly emphasizes identifying and visualizing trends, research collaboration relationships, and explosive growth of keywords within the scientific literature. VOSviewer, on the other hand, is used to create and visualize networks based on scientific literature, such as citation, co-citation, and co-authorship networks. In this article, VOSviewer is mainly applied to analyze Author collaboration network density maps and Network diagrams of keyword co-occurrence, revealing collaboration relationships among researchers and presenting keywords through network diagrams to analyze their strength of connection and structure, thereby identifying dominant themes and potential research gaps in the field. Through the integrated use of CiteSpace 6.2R6 software and VOSviewer 1.6.20, researchers can gain a comprehensive understanding from a macro perspective of research trends and network structures in acupuncture treatment for sciatic nerve injury. This approach effectively uncovers core themes, key nodes, and technological evolution within the research domain, assisting researchers in understanding the knowledge structure, current development status, research hotspots, comprehensive application prospects, and limitations in this field, aiming to guide current and future practitioners of SNI therapy.

## 2 Data and methods

### 2.1 Data source

The search period was defined as spanning from January 1, 2014, to December 31, 2023. The Chinese language was utilized in an advanced search mode, with the subject term search strategy comprising the terms “acupuncture” “electroacupuncture” and “acupoint.” The Web of Science database selected the WOS core set, and the specific search strategy was TS = (“Sciatic nerve injury” OR “Sciatic nerve”) AND (“acupuncture” OR “electroacupuncture “OR “moxibustion”).

### 2.2 Inclusion criteria

(1) The language of inclusion in the Chinese database was Chinese, while the language of inclusion in the WOS database was English. (2) The type of study included in the Chinese database was “Animal Experimentation, Clinical Research, Review,” while in the English database, it was “Article OR Review Article.” (3) All literature pertinent to the search terms was included, and the full text was available. (4) The main treatment measures of acupuncture were used to treat SNI, either alone or in combination with other therapies.

### 2.3 Exclusion criteria

The following sources were excluded:

1. Dissertations, conference papers, scientific, and technological achievements, newspapers, and research outputs.
2. Duplicate publications and articles with incomplete information.

### 2.4 Literature screening

The Chinese literature retrieved from CNKI,^1^ Wanfang,^2^ and VIP^3^ was imported into NoteExpress software for screening. This was done according to the inclusion and exclusion criteria. Once the screening was complete, the Chinese literature was confirmed for inclusion and exported in Refworks-CiteSpace format. Similarly, according to the established inclusion and exclusion criteria, the English literature from WOS that met the aforementioned criteria was identified and excluded. This literature was then downloaded and saved in the “full record and cited references” and “plain text” formats.

### 2.5 Data analysis

The saved Chinese and English documents were named in the format “download_x-x.txt,” imported into CiteSpace software, and de-emphasized again. The time segment of CiteSpace was selected as “2014–2023,” and the time partition defaulted to “1.” The time segment of CiteSpace was selected as “2014–2023,” the default time segment was “1,” and the parameter of threshold was set as “Top *N* = 50.” This data was transformed into a recognizable format by the CiteSpace converter and subjected to visual analysis in terms of the number of publications, institutions, authors, and keywords. To identify the most relevant core authors and keywords, VOSviewer software was employed for analysis. The analysis type was set to co-authorship and co-occurrence, the analysis unit was selected for authors and keywords, and the calculation method was selected to full counting. The visualization mapping was obtained by running the software, and the thresholds, sizes, colors of nodes, labels, and the clarity of connecting colors were adjusted manually to enhance the clarity and beauty of the resulting images.

## 3 Results

### 3.1 Results of the literature search

**Figure 1:**
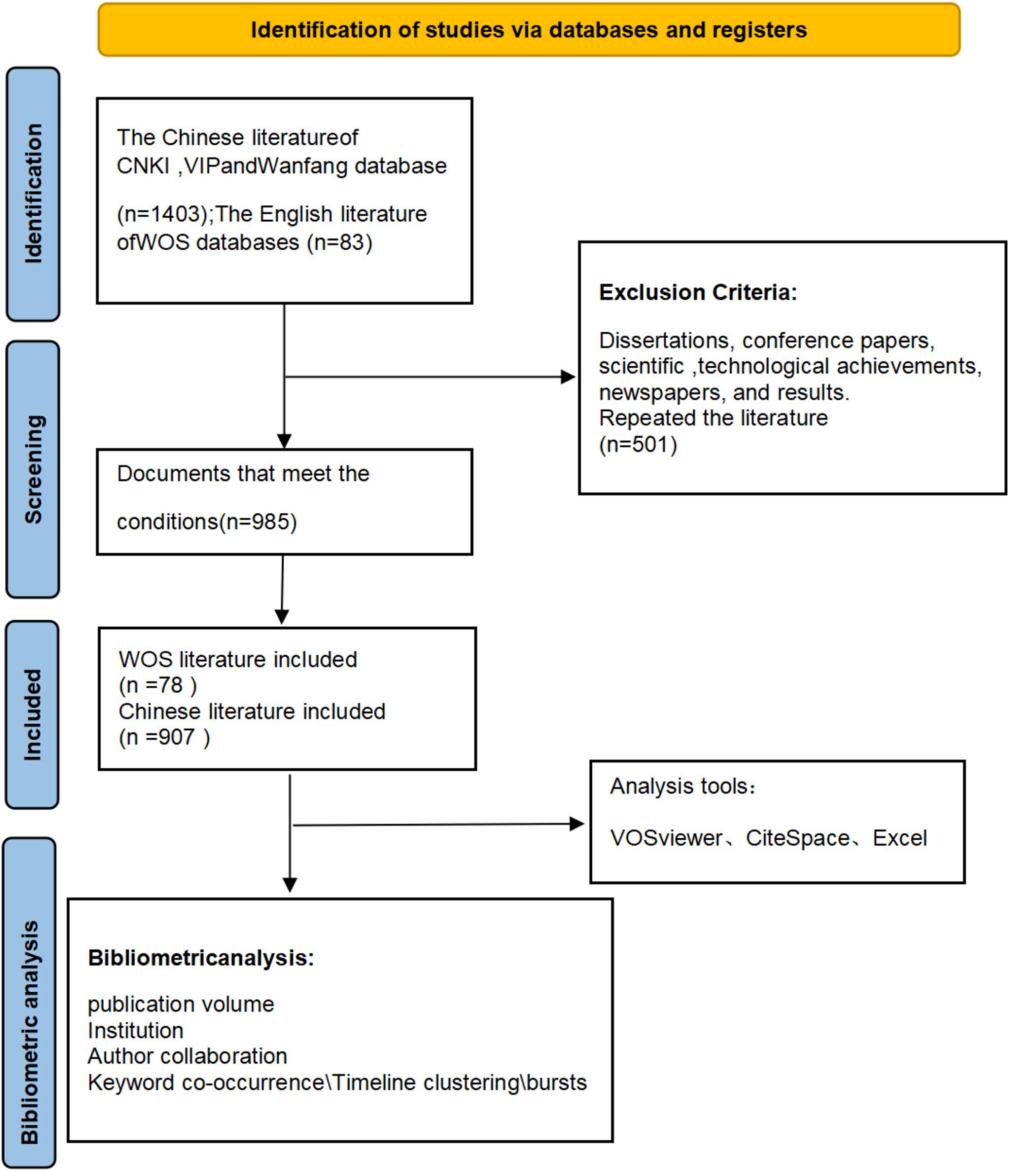
Flow chart of literature selection.

A total of 1,403 Chinese literature sources were included in the initial screening according to the exclusion criteria. Of these, 240 were from CNKI, 336 were from VIP, and 827 were from Wanfang. After applying the exclusion criteria and conducting a full-text review, 907 Chinese literature sources were included in the final analysis. Similarly, 78 English literature sources were included after applying the exclusion criteria and conducting a full-text review. This full process is outlined in the PRISMA flowsheet (Figure 1).

### 3.2 Visual analysis of publication volume

**Figure 2:**
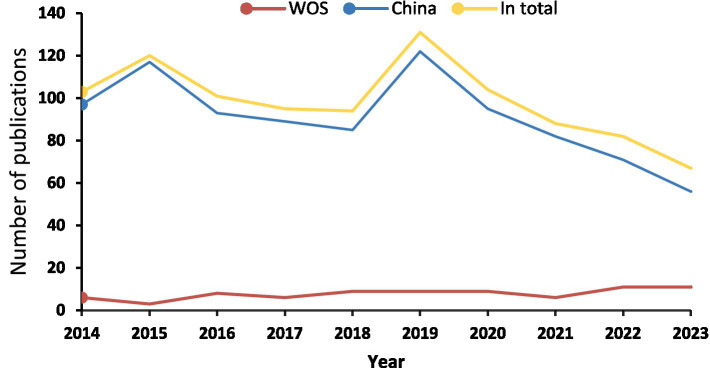
The number of publications on sciatic nerve injury treated by acupuncture from 2014 to 2023.

The included literature was subjected to statistical analysis in terms of the number of publications according to the time of publication, as illustrated in Figure 2. The number of English literature publications in the past 10 years is 78, with a relatively stable overall development trend. The highest number of issuances was recorded in 2022 and 2023, with 11 publications each year. The number of Chinese literature publications is 907, with a total number of issuances that shows an upward trend. The number of articles issued exhibited a downward trend from 2014 to 2015, but then rose rapidly after a slow decline from 2015 to 2018, reaching a peak in 2019. From 2019 to 2023, the number of articles issued decreased year by year.

### 3.3 Visualization of institutional analysis

**Figure 3:**
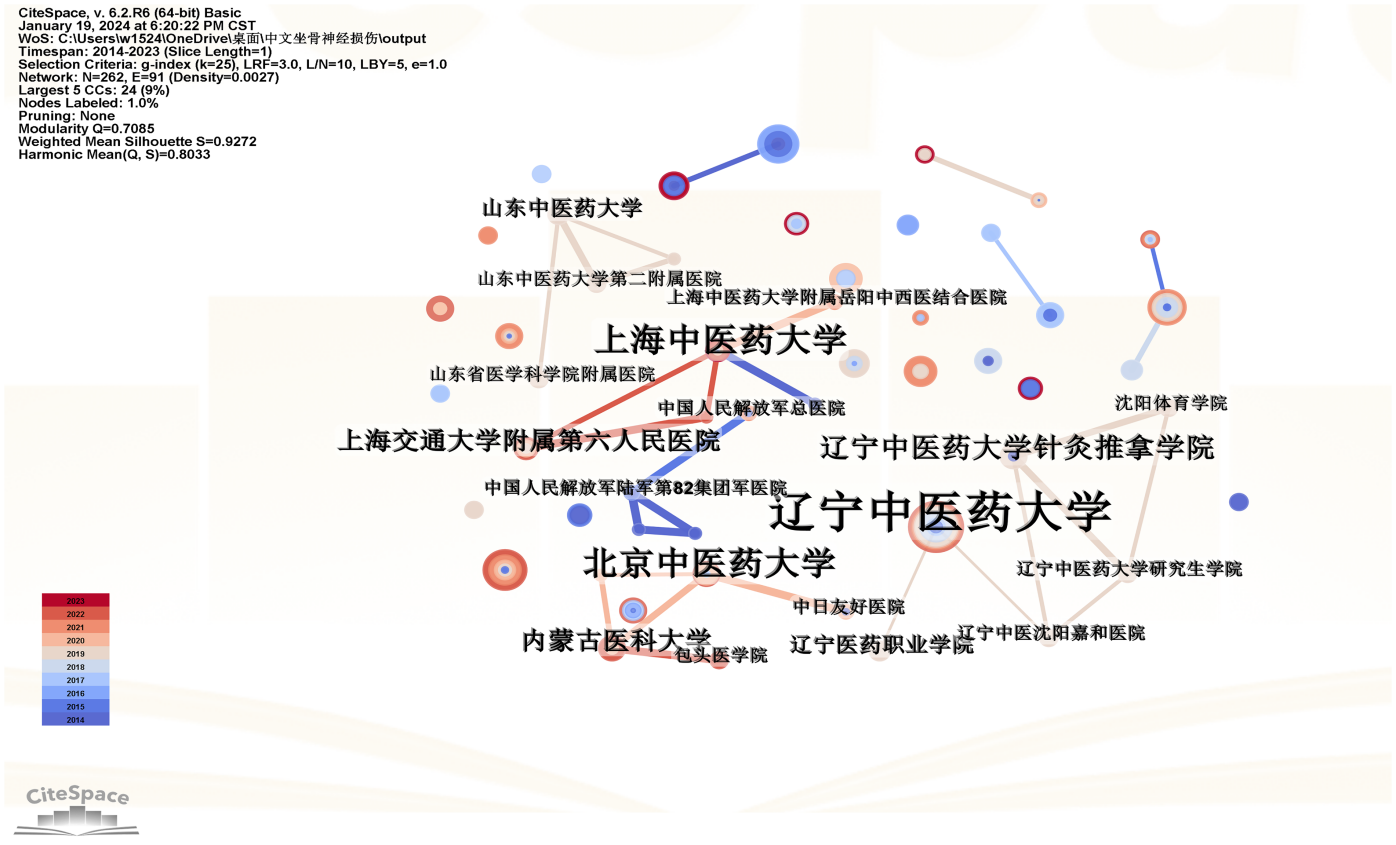
Co-occurrence map of the cooperative network of Chinese database institutions. Each node represents an institution, the size of the circles is proportional to the volume of publications, the size of the purple circles is proportional to the centrality, and the thickness of the lines represents the closeness of the links between institutions.

**Figure 4:**
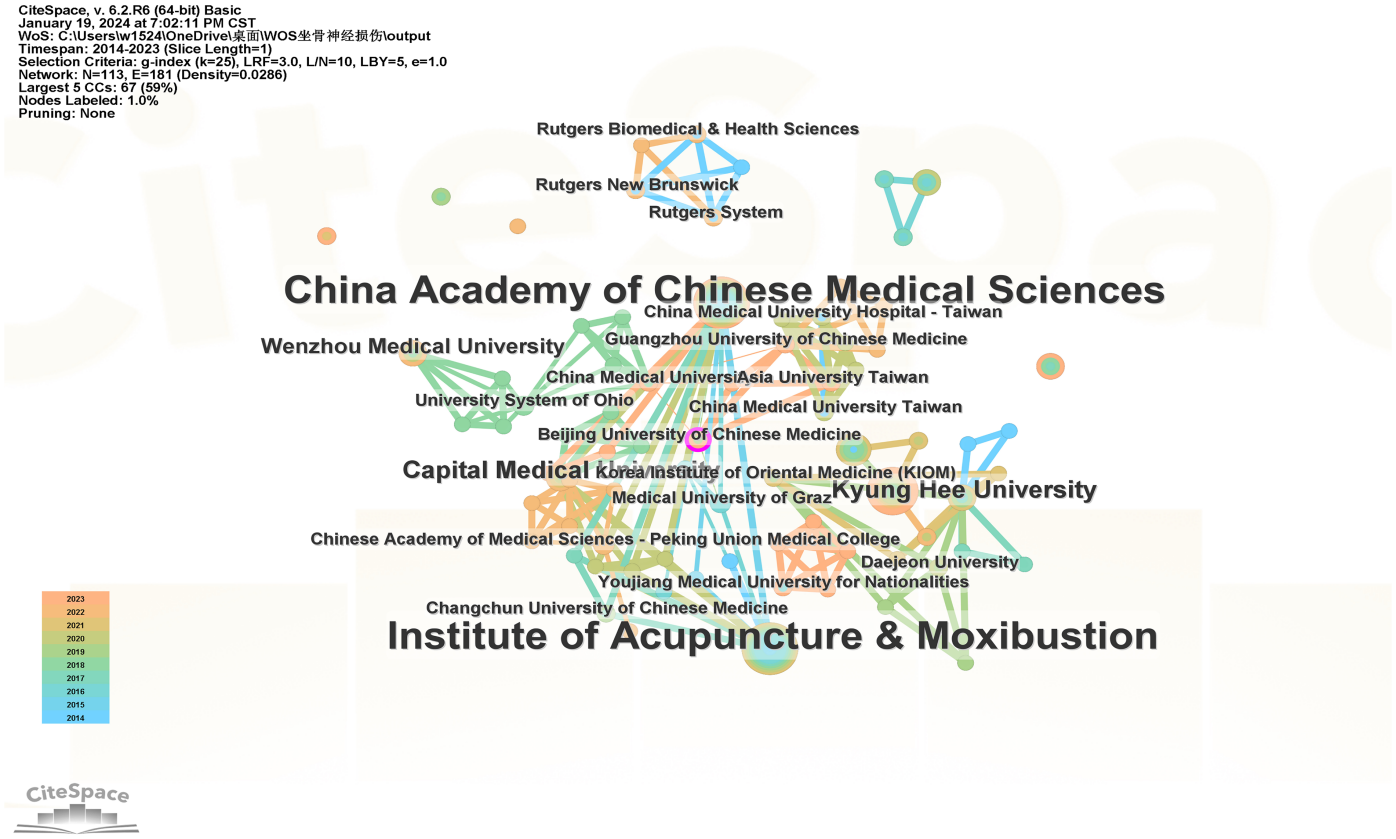
Co-occurrence map of the WOS database organization cooperative network. Each node represents an institution, the bigger the node the institution had more publications. The line indicated the relationship between institutions, the thicker the closer the relationship, the lighter the later cooperation was formed.

Research on Chinese databases has found that 262 institutions have published articles in this field, with universities being the main hubs of research. The top three institutions in terms of publication volume are Liaoning University of Traditional Chinese Medicine (辽宁中医药大学)20 articles, Heilongjiang University of Chinese Medicine (黑龙江中医药大学) 13 articles, and Fujian University of Traditional Chinese Medicine (福建中医药大学) 10 articles. Meanwhile, the WOS database shows that 113 institutions have published relevant articles, with the top three being the Acupuncture Research Institute (针灸研究所) 12 articles, the Chinese Academy of Medical Sciences (中国医学科学院) 12 articles, and the Shanghai University of Traditional Chinese Medicine (上海中医药大学) 11 articles. Using CiteSpace software for mapping and analysis, as shown in Figures 3, 4, the Chinese database displays 262 nodes with 91 links, resulting in a network density of 0.0027, indicating loose connections among institutions. Most collaborations are concentrated among universities, their affiliated hospitals, and colleges, with less inter-provincial and cross-disciplinary cooperation. The visualization from the WOS database shows 113 nodes and 181 links, with a network density of 0.0286. Research in this field is mainly concentrated among various institutions in China, and domestic collaborations are relatively close. However, the centrality of institutions in both databases is low, suggesting that this field has yet to develop high-impact institutions.

### 3.4 Author collaboration visualization analysis

**Figure 5:**
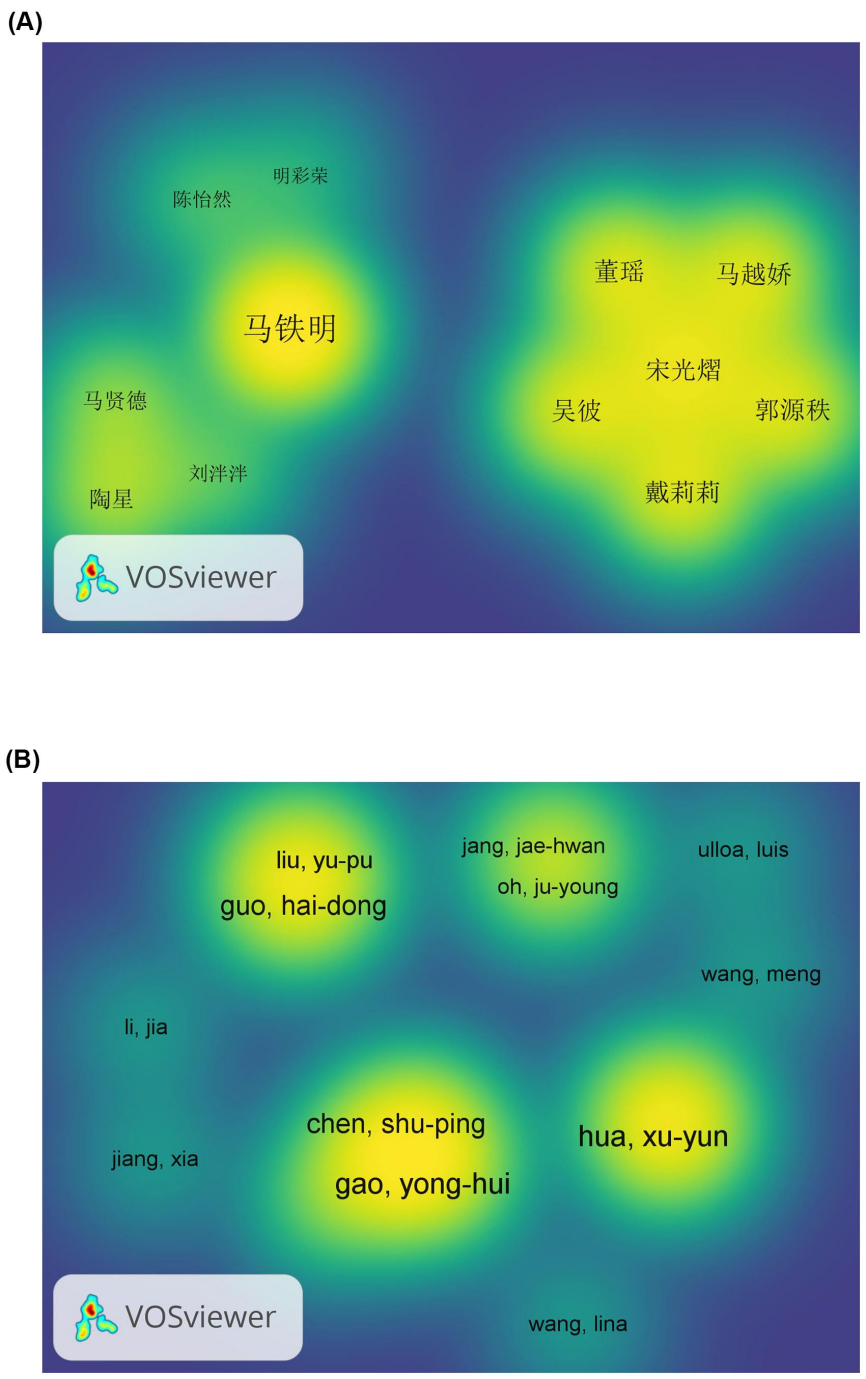
**(A,B)** Author collaboration network density map of the Chinese database and WOS database. The higher the author density, the closer the color is to bright yellow. Smaller densities tend to be blue. The density depends on the strength and importance of the author’s connections in the surrounding area.

A total of 10 authors with a minimum of seven published works in the Chinese database were identified. These authors are presented in Table 1. The highest-ranking author in terms of both the number of published works (32) and the strength of the connections (38) is Ma Tie Ming. In contrast, Wang Yan and Aghul are connected to the other authors with a strength of 0, indicating a relatively weak connection. According to the principle of Pareto’s law, the number of published works (M) can be calculated using the following formula: M = 0.749 × (Nmax). The mean of the authors’ publication rates was calculated, resulting in M = 0.749 × 32 1/2 ≈ 4. This indicates that authors with a publication rate of at least 4 articles are considered the core contributors to the field, with a total of 85 individuals. The VOSviewer software was utilized to create a co-authorship density map, which allows for a clear visualization of the relationships between authors with high influence and the density of their collaborations. The weights were set to Total link strength, as illustrated in Figure 5A. A total of 907 Chinese-language documents were linked to 3,435 authors, with the network diagram displaying authors with a minimum of four publications. This research area has been divided into three research teams, with the largest comprising four members. To enhance clarity, the diagram has been simplified. It should be noted that the team members are not displayed in their entirety. In the WOS database, the author with the highest number of publications is Gao Yonghui, with six other authors involved, namely Hua Xuyun, Liu Junling, Wang Junying, Wu Jiajia, and Xu Jianguang. According to the Pareto principle, M ≈ 2, the WOS database encompasses 523 authors. The minimum number of published articles was set at two, and the resulting list of authors with at least two publications is shown in Figure 5B. This yielded 37 core authors, who formed a team centered around Guo Hai-dong, Chen Shu-ping, Jang Jae-hwan, and Xu Jian-guang. The team members were typically from the same university or affiliated hospital, or the same region.

**Table 1:** Top 10 authors in the number of Chinese database publications.

Rank	Name	Publications	Total link strength
1	Ma Tieming (马铁明)	32	38
2	Tang Chenglin (唐成林)	9	36
3	Fang Jianqiao (方剑桥)	9	17
4	Wang Zhifu (王志福)	8	10
5	Wang Yan (王艳)	8	0
6	Du Junying (杜俊英)	7	15
7	Wang Hongfeng (王洪峰)	7	10
8	Zhao Dan-dan (赵丹丹)	7	32
9	Aaqoura (阿古拉)	7	0
10	Chen Yiran (陈怡然)	7	7

## 4 Keywords

### 4.1 Keyword co-occurrence visualization analysis

**Figure 6:**
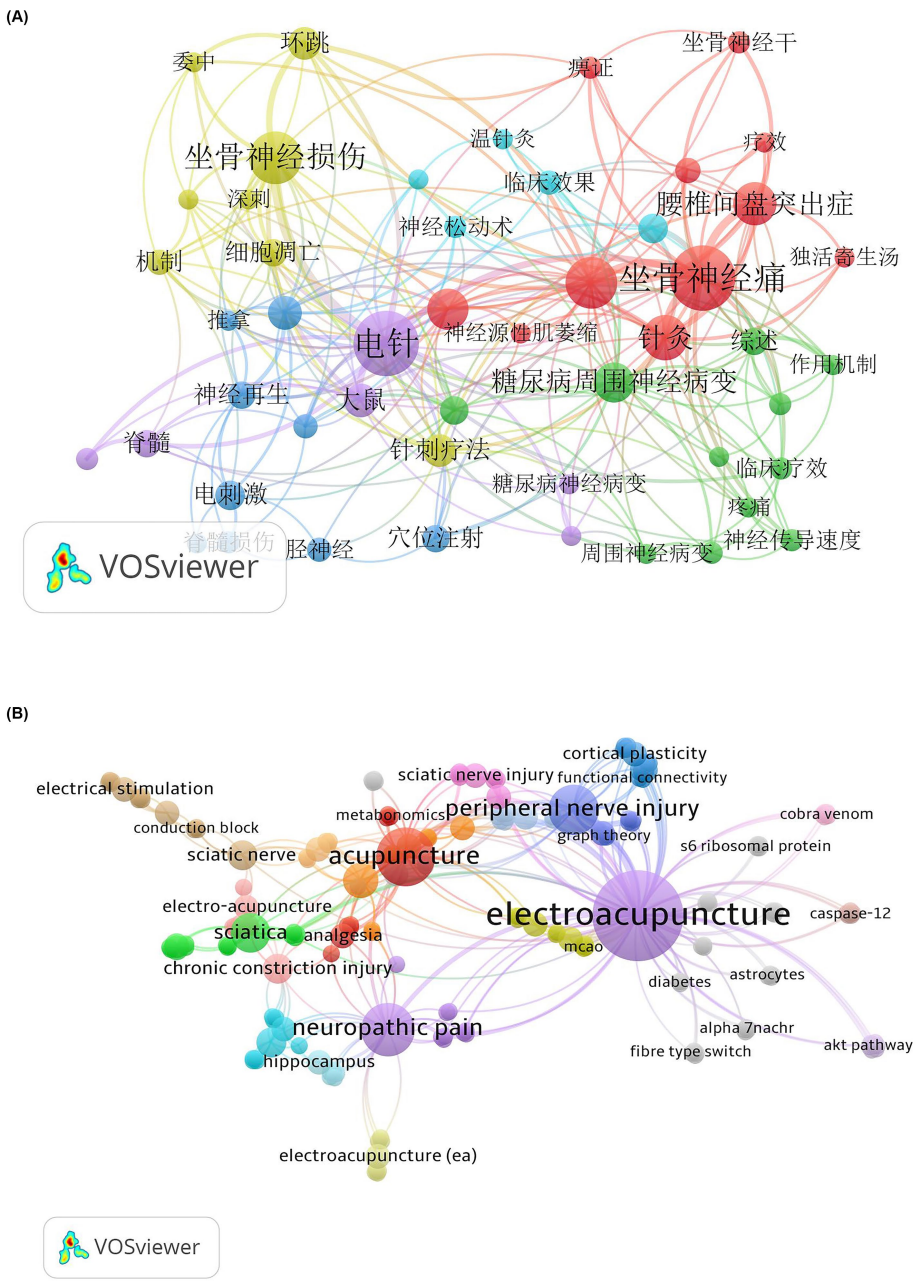
**(A,B)** Network diagram of keyword co-occurrence between Chinese database and WOS database. The shows the network diagram of the Co-occurrence analysis of keywords. The circles represent keywords, and the size of the circles represents the frequency of keywords. The size of the circles is proportional to the frequency of keywords. Circles with the same color form a cluster group, and the connecting lines represent the connection between keywords.

In bibliometrics, the frequency of keywords appearing in a given study area can reveal the prevailing research interests and trends within that field. By analyzing the keywords in the Chinese database and the WOS database using the CiteSpace software, we found that the two databases included 1,256 and 227 keywords, respectively. The top 10 keywords with the highest occurrence frequency are presented in the following table. Tables 2, 3 demonstrate that in the Chinese database, the most central keyword is electroacupuncture (0.50), while in the WOS database, the most central keyword is neuropathic pain (0.36). The keyword co-occurrence maps were created using VOSviewer, as shown in Figures 6A,B. Analysis of both Chinese and English keywords revealed the following: (1) Electroacupuncture is the primary intervention method for acupuncture, with other methods including moxibustion (艾灸), herbal moxibustion (草药灸), and needle-knife therapy (针刀); (2) Besides using acupuncture alone to treat SNI-related diseases, it is often combined with massage (按摩), electrical stimulation (电刺激), nerve loosening techniques (神经松动术), acupoint injection (穴位注射), and traditional Chinese medicine (中药); (3) Causes of SNI include piriformis syndrome (梨状肌综合征), lumbar disc herniation (腰椎间盘突出症), spinal cord injury (脊髓损伤), chronic compressive injury (慢性压迫性损伤), and diabetic peripheral neuropathy (糖尿病周围神经病变); (4) The main acupoints targeted for this disease are the Weizhong (委中) and Huantiao points (环跳); (5) SNI often leads to neurogenic muscle atrophy (神经源性肌萎缩), neuropathic pain (神经性疼痛), and inflammation (炎症); (6) Molecules and related regulatory pathways involved in nerve repair include Schwann cells (雪旺细胞), nerve growth factor (神经生长因子), brain-derived neurotrophic factor (脑源性神经生长因子), apoptosis (细胞凋亡), and the Akt pathway (Akt通路); (7) Evaluation methods include sciatic nerve conduction velocity (坐骨神经传导速度) and functional index (功能指标).

**Table 2:** Frequency and centrality of keywords in Chinese database.

Rank	Keyword	Frequency	Centrality
1	Electroacupuncture	70	0.52
2	Sciatica	69	0.33
3	Acupuncture	41	0.36
4	Acupuncture and moxibustion	24	0.12
5	Sciatic nerve	22	0.28
6	Rat	14	0.02
7	Huan tiao	13	0.04
7	Lumbar disc herniation	9	0.10
8	Electrical stimulation	7	0.18
9	Clinical effect	6	0.03
10	Spinal cord	5	0.02

**Table 3:** Occurrence frequency and centrality of keywords in the WOS database.

Rank	Keyword	Frequency	Centrality
1	Electroacupuncture	26	0.24
2	Neuropathic pain	17	0.36
3	Peripheral nerve injury	13	0.11
4	Expression	12	0.35
5	Activation	11	0.23
6	Mechanisms	8	0.05
7	Regeneration	8	0.04
8	Spinal cord	7	0.02
9	Electrical stimulation	5	0.1
10	Model	5	0

### 4.2 Keyword cluster visualization analysis

The CiteSpace visual analysis software was utilized to conduct a network analysis of keywords, resulting in the generation of a keyword cluster map. The log-likelihood ratio (LLS) algorithm was employed to identify eight Chinese keyword clusters and 10 English keyword clusters. It is generally accepted that S > 0.7 represents a convincing clustering structure. The mean silhouette S for the Chinese graph is 0.9272, while the mean silhouette S for the WOS graph is 0.851.This indicates that the keywords in the Chinese graph are more homogeneous than those in the WOS graph. The homogeneity of the data and the optimal performance of the graph are evident. The specific Silhouette values and labels (LLR) are presented in Tables 4, 5.

**Table 4:** Cluster information table of Chinese keywords.

Cluster ID	Size	Silhouette	Representative terms (LLR)
#0	30	0.848	Acupuncture; review; mechanism of action; small needle; lumbar disc herniation
#1	27	0.958	Electroacupuncture; rat; oxaliplatin; acupuncture; sciatic nerve
#2	24	0.927	Sciatic nerve; apoptosis; deep thorns; electroacupuncture; huantiao
#3	22	0.93	Acupuncture; diabetes; clinical efficacy; nerve conduction velocity; electroacupuncture
#4	21	0.866	Electrical stimulation; nerve regeneration; autophagy; peripheral nerves; Biomechanics
#5	19	0.903	Acupuncture; Neuralgia; rehabilitation; Sciatica; Chinese medicine fumigation
#6	18	0.893	Paralysis; Huan tiao; Hole; Nerve; Bending test
#7	10	0.91	Mechanism; spinal cord; inflammatory pain; ciliary neurotrophic factor; comparison

**Table 5:** Cluster information table of Chinese keywords.

Cluster ID	Size	Silhouette	Representative terms (LLR)
#0	33	0.837	Glucose metabolism
#1	26	0.891	Acupuncture analgesia
#2	25	0.764	Therapeutic effect
#3	23	0.791	Electroacupuncture therapy
#4	20	0.844	Low-frequency electroacupuncture
#5	18	0.898	Neuropathic rat
#6	17	0.916	Asymmetrical responses
#7	16	0.943	NK cell
#8	11	0.859	Acute sciatic nerve injury
#9	9	0.985	Electroacupuncture analgesia

**Figure 7:**
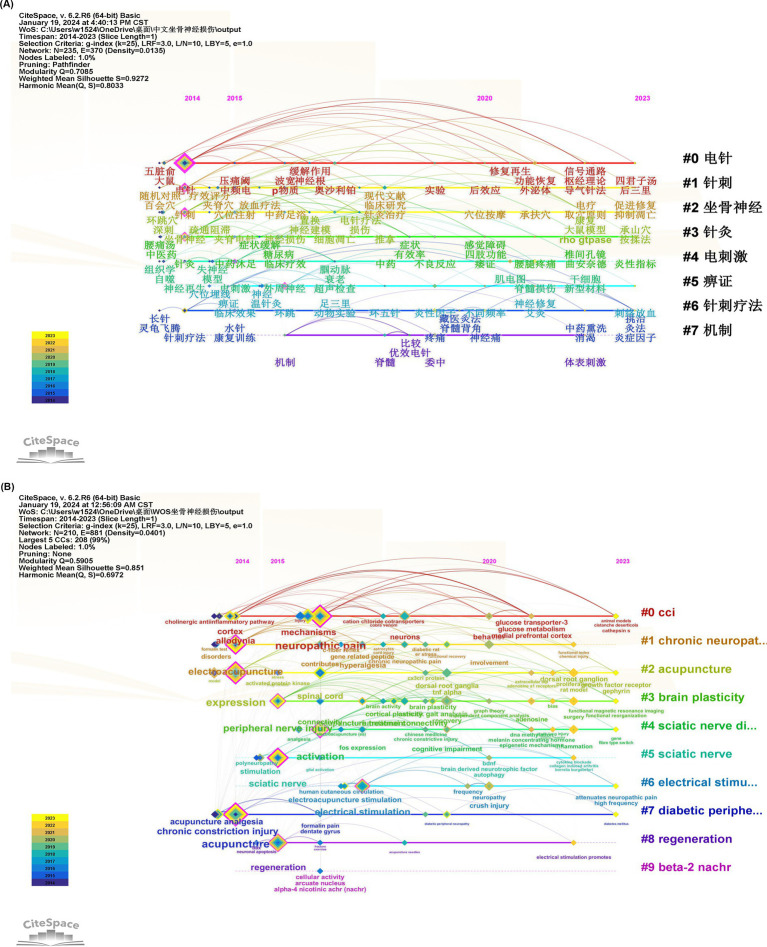
**(A,B)** Keyword clustering diagram of Chinese database and WOS database timeline. Each node in the timeline represents a keyword, and the closer the node is to the front, the earlier the keyword appears. Keywords in each timeline form a keyword cluster, and the cluster number is inversely proportional to the cluster size, such as#0 represents the largest cluster.

Timeline clustering maps can visually display keywords in chronological order, revealing the hotspots and phase characteristics of keywords in related fields (34). By running the CiteSpace visualization software for keyword timeline mapping analysis, Figures 7A,B respectively show the distribution of keyword timelines across Chinese and English databases. In the Chinese timeline clustering map, keywords such as acupuncture and electroacupuncture appeared early and have a wide timespan, playing a dominant role in the repair of SNI. In recent years, new treatment methods such as guided-qi acupuncture (导气针法), picking therapy (挑治), bloodletting acupuncture (刺络放血), traditional Chinese medicine fumigation (中药熏洗), moxibustion (灸法), and acupressure (按揉法) have emerged. Huantiao (环跳), Weizhong (委中), and Jiaji points (夹脊) are commonly used acupuncture points for treating SNI, and in the past 2 years, researchers have chosen Housanli (后三里) and Chengshan points (承山) for diagnosis and treatment. In the WOS timeline clustering map, keywords such as acupuncture, electroacupuncture, neuropathic pain, expression, and acupuncture analgesia were found earliest and continue to this day, with keywords like neuropathic pain, activation, and mechanisms emerging in 2016 and persisting to the present.

### 4.3 Keyword with the strongest citation bursts visualization

**Figure 8:**
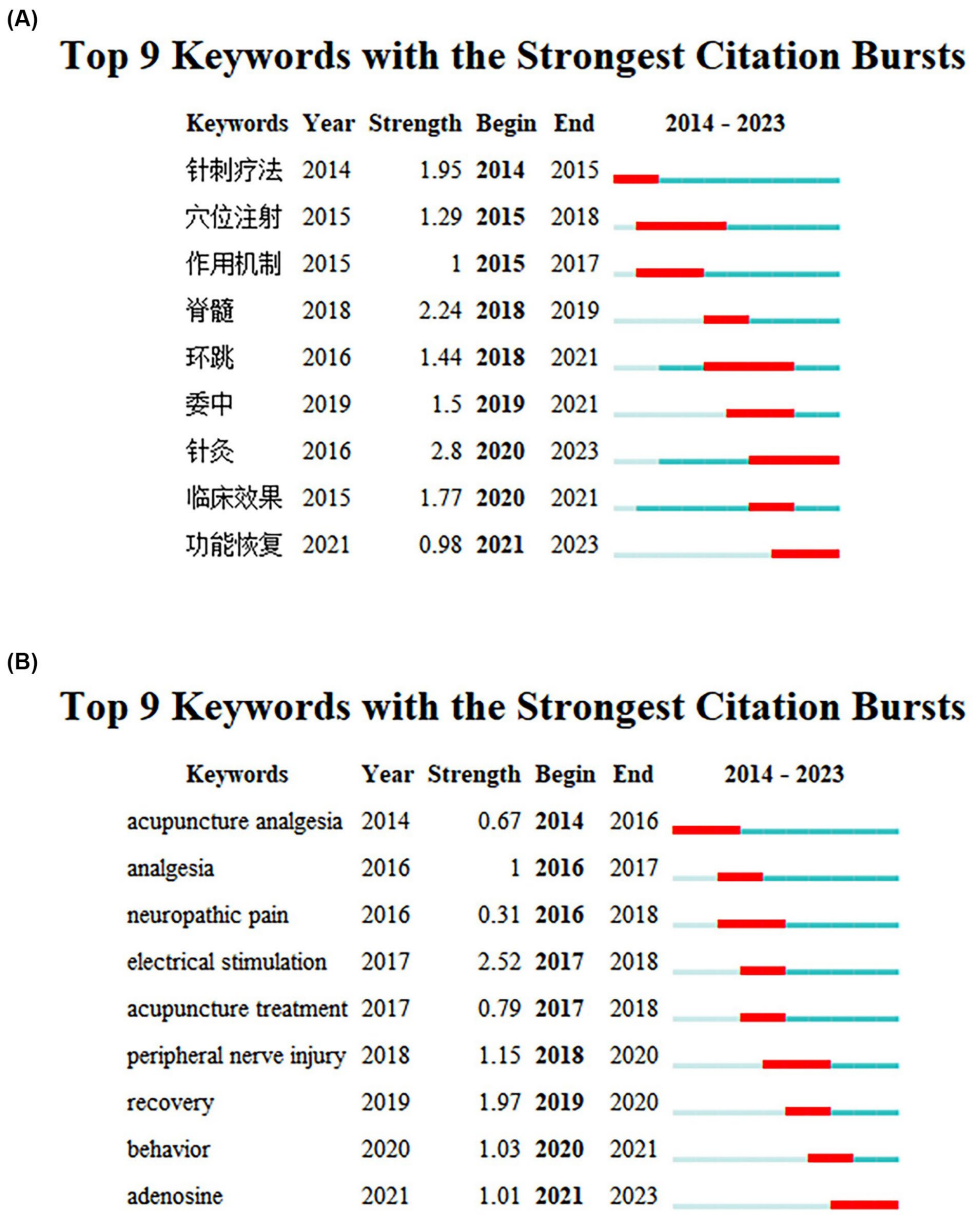
**(A,B)** The diagram shows the top nine keywords with the strongest citation bursts of Chinese database and WOS database. The blue lines indicate the time intervals, and the red lines indicate the duration of the citation bursts.

The CiteSpace software was employed to conduct a co-occurrence analysis of the selected literature, which identifies the most frequently occurring terms within a specific temporal range. This analysis can be utilized to discern the prevailing trends within a given research domain. Figures 8A,B illustrate the top nine keywords with the highest citation frequency in the published literature on acupuncture for SNI-related treatments. The blue lines represent time intervals, while the red lines represent the duration of the cited references. The overall strength of the database keywords is relatively low, with a brief duration of citation. The Chinese database keywords map shows that acupuncture therapy was first documented, with a strong spinal cord (脊髓) citation (2.24). Other prominent keywords include acupoint injection (穴位注射), mechanisms of action (作用机制), weizhong (委中), huantiao (环跳), clinical effect (临床效果), acupuncture (针灸), and functional recovery (功能恢复). Notably, acupuncture and functional recovery have emerged as major research topics in recent years and have maintained their prominence. In contrast, the WOS database keywords map shows that acupuncture for pain relief was the earliest documented keyword, with the highest citation strength (1.97). Other prominent keywords include pain, neuropathic pain, electrical stimulation, acupuncture, peripheral nerve injury, and behavior. These findings suggest that acupuncture plays a crucial role in SNI pain management. Adenosine is a recently emerging keyword, indicating its potential as a promising treatment for SNI-induced pain. The keyword “pain” was the earliest to emerge, with the highest intensity of occurrence (1.97). Other keywords of particular interest include “pain management,” “neuropathic pain,” “electrical stimulation,” “acupuncture,” “peripheral nerve injury,” and “behavior.” These findings suggest that acupuncture plays a significant role in SNI pain management. Adenosine was the most recently identified keyword, with involvement in sciatic nerve repair. Research indicates that adenosine 2A receptor agonists improve motor function in rats with SNI and relieve heat pain sensitivity (7).

## 5 Discussion

### 5.1 Bibliometric analysis

This study utilizes CiteSpace and VOSviewer to conduct a visual analysis of the Web of Science database. The resulting visualizations include a bibliometric map featuring the most cited authors, institutions, countries, and keywords. Data analysis indicates that acupuncture has significantly captured the interest of Chinese scholars, marked by a substantial increase in publications in both Chinese and English over the past decade. A total of 85 articles were published in 2015, with peaks of 120 in 2019 and 137 articles, respectively. Although there was a slight decline in the number of articles published between 2019 and 2023, the overall annual publication rate consistently exceeded 67 articles. This suggests that the field will continue to be a key research focus in the future. The majority of the publishing institutions are located in China, with the top institutions being Liaoning University of Traditional Chinese Medicine, Heilongjiang University of Chinese Medicine, and Fujian University of Traditional Chinese Medicine. Notably, Heilongjiang University of Chinese Medicine, the second-highest-ranking institution, distinguishes itself from others in the network, indicating a need for greater interdisciplinary and interregional collaboration. The Acupuncture Research Institute is the most prolific publisher, maintaining robust connections with other institutions. However, the overall influence of these institutions remains limited due to their relatively weak centrality. The study identifies 12 Chinese-language core teams and six English-language core teams. Ma Feiming, the author with the highest publication output, has published 32 articles and established strong connections, demonstrating a significant influence in the field. His primary research focuses on acupuncture at the “Huantiao “and “Weizhong” points and their roles in restoring SNI mechanisms (8–13).

### 5.2 Research highlights and outlook

#### 5.2.1 Selecting acupoints for acupuncture in SNI

The efficacy of acupuncture in SNI hinges on the selection of acupoints. The most commonly used acupoints include the following:

The relationship between the acupoints of the foot and the acupuncture meridians has been demonstrated to have a positive effect on the treatment of SNI (14). This is thought to be due to the stimulation of energy flow and nerve stimulation, which in turn has a beneficial effect on the condition (15). The acupoint of The “Zusanli” or “Weizhong” acupoint is linked to the spleen and stomach meridians. It is thought that this acupoint may have a role in regulating the balance of moisture and heat within the body, as well as reducing the inflammatory response. This in turn may facilitate the regeneration of nerves. Clinically, this acupoint is often used alongside the “Yanglingquan” acupoint on the lower back. This combination of acupoints has been shown to improve the function of the sciatic nerve and the transmission of nerve signals. This in turn may facilitate the repair of damaged nerves (13). Jiaji points are a type of extra meridian point in traditional Chinese medicine. Stimulation of Jiaji points can have a regulatory effect on the nervous system, helping to alleviate inflammation and relieve pain in cases of SNI by modulating neural excitability and preventing secondary neuronal damage. Studies have shown that, following intervention with electroacupuncture at Jiaji points combined with nerve mobilization techniques in SNI-induced rabbits, improvements were observed in muscle strength and sciatic nerve function (16).

### 5.3 Mechanisms of action of acupuncture in SNI

The mechanisms of action of acupuncture in SNI may involve the proliferation of Schwann cells, the secretion of neurotrophic factors (NTFs) and extracellular vesicles, and the expression of neural cell adhesion molecules (NCAM) and ciliary neurotrophic factor (CNTF) (13, 17). Studies have demonstrated that acupuncture may enhance the formation of new axonal connections in the surrounding nerves, promote the proliferation of Schwann cells, and facilitate the secretion of NCAM and CNTF, thus exerting neuroprotective effects, alleviating pain, and promoting the regeneration of myelin and functional recovery (18, 19). Acupuncture may also act through the activation of the PI3K/Akt/mTOR signaling pathway or the modulation of interhemispheric functional connections and directional effects, thereby promoting the recovery of motor function in SNI (20, 21). Furthermore, in order to gain a deeper understanding of the potential mechanisms underlying the efficacy of acupuncture in the treatment of diseases, numerous researchers have focused their attention on the involvement of IL-1β, IL-6, and TNF-*α* in the inflammatory response and the expression of NF-κB protein, which has led to the discovery that electroacupuncture can effectively inhibit the inflammatory response and NF-κB protein expression, thereby accelerating the pathological repair process (22–25). Pain is a significant challenge in the context of SNI. In their studies, researchers have demonstrated that electroacupuncture can relieve pain by inhibiting ERK and p38MAPK signaling pathways and upregulating *γ*-aminobutyric acid (GABA) receptors. It has been demonstrated that acupuncture can alleviate neuropathic pain and hyperalgesia (26, 27).

### 5.4 Interventions of acupuncture in SNI

Combining keywords, it is evident that in the selection of acupuncture therapy, the intervention methods mainly include electroacupuncture, acupoint injection, warm acupuncture, moxibustion, etc. Among them, electroacupuncture is the most widely used intervention method in this field. The rhythmic vibration of electroacupuncture can accelerate muscle fiber contraction, promote local tissue microcirculation, and improve the state of muscle atrophy (35). Additionally, many other intervention methods are also being researched and focused on by scholars, such as needle knife therapy. Some researchers have found that needle knives can regulate central analgesic substances such as 5-hydroxytryptamine, *β*-endorphin, and norepinephrine to achieve analgesic effects (28, 29). In combining other treatment methods, more emphasis is placed on the importance of the comprehensive application of multiple treatment methods to improve treatment effectiveness. Combining massage, pharmacotherapy, physical therapy, nerve loosening, nerve transplantation, and rehabilitation training can comprehensively enhance treatment effects. For example, combining acupuncture with drugs can enhance the conduction velocity of the sciatic nerve, improve hyperalgesia symptoms, and control inflammation (30, 31). Electroacupuncture combined with massage significantly alleviates sciatic nerve pain caused by lumbar disc herniation and improves the limitation of physical activity (32). Zhang et al. (33) found that acupuncture at “Zusanli” and “Huantiao” points combined with rehabilitation training effectively promotes microvascular generation in sciatic nerve compression rats, which is beneficial for promoting nerve regeneration and functional recovery. It is clear that by integrating different treatment methods, a more comprehensive and personalized SNI treatment plan can be achieved.

This study utilized CiteSpace and VOSviewer software to conduct a visualization analysis of literature in the Web of Science database, highlighting the role of acupuncture in the repair and regeneration of SNI. The findings show that, over the past decade, there have been a total of 985 publications in both Chinese and English on this topic, with research hotspots primarily focused in China. Literature analysis indicates that institutions such as Liaoning University of Traditional Chinese Medicine have a high volume of publications in this area, while Heilongjiang University of Traditional Chinese Medicine stands alone without close connections to other institutions, underscoring the need for cross-disciplinary and cross-regional collaboration. In SNI treatment, the key aspect of acupuncture lies in point selection, with frequently used points such as Huantiao and Weizhong. Through modulation of neural excitability, inflammatory response, and neuroprotection, acupuncture promotes nerve regeneration and functional recovery. Additionally, the mechanisms of acupuncture involve Schwann cell proliferation, secretion of neurotrophic factors, and regulation of signaling pathways. Integrating different therapies such as electroacupuncture, moxibustion, and tuina can contribute to more comprehensive and personalized SNI treatment approaches. Future research should delve deeper into the molecular mechanisms and validate its efficacy on a larger clinical scale.

## 6 Conclusion

This study provides a comprehensive review of the research conducted over the past decade on acupuncture in the field of SNI. It highlights electroacupuncture and neuropathic pain as key emerging topics in this area. The research identifies commonly used acupuncture points and associated mechanisms of injury repair in the treatment of SNI. Acupuncture is often combined with pharmacotherapy, physical therapy, and rehabilitation training to enhance therapeutic efficacy. The mechanisms underlying acupuncture’s effects remain a focal point and challenge in current research. While acupuncture exerts its reparative effects on SNI through various mechanisms, further investigation is required to elucidate its specific molecular mechanisms and to validate its efficacy in larger-scale clinical practice. Additionally, exploring personalized acupuncture treatment strategies tailored to individual patient differences represents a promising direction for future research.

## 7 Limitations of the study

(1) The English database selects literature solely from the Web of Science Core Collection’s indexed journal database. As a result, the findings may be biased, and future searches could be expanded and explored more deeply. (2) There are inconsistencies in the terminology used in the keywords of published articles. For example, terms like “neuralgia,” “neuropathic pain,” and “sciatica” are not consistently defined or applied. (3) Moreover, the literature from China was sourced from multiple databases, which led to the exclusion of cited journals, literature, and authors when imported into the visualization software. This limitation hindered a comprehensive analysis of the included articles. (4) The majority of the included studies are animal experiments. Future research should focus on delving deeper into the physiological mechanisms of acupuncture points and validating the effectiveness of this treatment strategy through broader clinical practice.

## Data Availability

The datasets presented in this study can be found in online repositories. The names of the repository/repositories and accession number(s) can be found in the article/supplementary material.

## Footnotes

^1^ https://www.cnki.net/

^2^ https://www.wanfangdata.com.cn

^3^ https://qikan.cqvip.com/
